# Angiosarcoma of the breast: A review

**DOI:** 10.1016/j.heliyon.2024.e24413

**Published:** 2024-01-19

**Authors:** Ran An, Xiao-Juan Men, Xi-Hao Ni, Wei-Tao Wang, Chang-Liang Wang

**Affiliations:** a School of Clinical Medicine, Shandong Second Medical University, Weifang 261041, Shandong Province, China; b Department of Thyroid and Breast Surgery, Weifang People's Hospital, Weifang 261041, Shandong Province, China

**Keywords:** Breast angiosarcoma, Diagnosis, Treatment

## Abstract

Breast angiosarcoma is a rare and highly aggressive malignancy with a poor prognosis. It can occur spontaneously or be associated with factors such as radiation therapy or chronic lymphedema. The etiology and pathogenesis of this disease are still unclear, the clinical symptoms and imaging findings lack specificity, and the pathological morphology is diverse, which is easy to be confused with other diseases. There is no clear guideline for surgical treatment. Although the optimal surgical approach remains unclear, the ultimate goal is surgical excision with optimal margins, which remains the primary method of treatment. In clinical practice, the choice of the surgical approach should be made by considering the tumor size, pathological type, and patient preferences. In clinical practice, the selection of surgical methods should be carried out with comprehensive consideration of tumor size, pathological types and patients' wishes. There is no clear consensus on whether radiotherapy and chemotherapy should be carried out after surgery, and its optimal program and efficacy are uncertain. This article reviews the etiology, clinical manifestations, pathological features, imaging findings, treatment, prognosis and other aspects of breast angiosarcoma, so as to strengthen clinicians' overall understanding of this disease and avoid missed diagnosis and misdiagnosis.

Breast angiosarcoma (BA) is a relatively rare clinical condition, accounting for approximately 0.04 % of all malignant breast tumors [1]. It is a malignant tumor originating from the endothelium of the blood vessels surrounding breast lobules or within lobular capillaries. Based on etiology, BA can be classified into primary breast angiosarcoma (PBA) and secondary breast angiosarcoma (SBA). PBA is an aggressive and poorly prognostic tumor, usually diagnosed in women aged 30–50 without a history of cancer or identifiable risk factors [2]. Conversely, SBA tends to affect older women, with a median age of 67–71 years, and is often linked to prior radiotherapy [3]. Reports of radiation-induced angiosarcoma have increased with the rise in breast-conserving surgeries followed by radiation therapy [4]. Additionally, SBA is also linked to chronic lymphedema following radical mastectomy for breast cancer (also known as Stewart-Treves syndrome) [5]. Current research mainly consists of case reports and small-scale single-center retrospective analyses, resulting in limited sample sizes. This article provides a comprehensive review of the current research status on the etiology, clinical presentation, pathological characteristics, imaging findings, treatment, prognosis, and other aspects of BA.

## 1 Etiology

Due to the rarity of BA, its etiology and pathogenic mechanisms remain unclear. Most viewpoints suggest associations with factors such as chronic lymphedema, exposure to chemical substances, ionizing radiation, chronic infections, and traumas, but there is no explicit data supporting this claim [6]. Additionally, 6 %–12 % of BA cases occur in pregnant women, with higher incidence rates during pregnancy and lactation periods. This is hypothesized to be linked to elevated estrogen levels, although there is ongoing debate [7]. In the context of 16 documented instances of pregnancy-related angiosarcoma, the potential influence of hormonal triggers is hypothesized, given the manifestation of the tumor in a younger age cohort [8]. Furthermore, Brentani et al. described the existence of estrogen and glucocorticoid receptors in BA [9], but definitive literature confirming their relevance is lacking. Meanwhile, as breast-conserving surgeries have advanced, there has been a growing number of reports concerning postoperative radiation therapy-associated BA [4].

## 2 Clinical presentation

The clinical presentation of this condition lacks specificity. In cases of PBA, patients typically present with rapidly growing, painless masses (≥4 cm), occasionally accompanied by rare instances of purplish-blue skin discoloration, often observed in young females. Conversely, SBA predominantly affects elderly women and is characterized by rashes, ecchymosis, or skin thickening near previous surgical sites. This is frequently accompanied by purplish-blue skin discoloration and tends to emerge after radiation therapy [10]. Clinically, secondary angiosarcomas, particularly those induced by radiation, involve the breast's dermis, occasionally extending into the breast parenchyma. This stands in contrast to primary angiosarcomas, which mainly develop within the breast parenchyma and only lead to skin changes after affecting the skin. Furthermore, reports have indicated that larger tumors can trigger thrombocytopenia and bleeding tendencies, recognized as the Kasabach-Merritt syndrome [11]. Due to its diverse clinical manifestations, close integration of pathological findings is essential in clinical practice. Attention to differential diagnoses with other diseases is crucial to avoid misdiagnosis or oversight.

## 3 Pathological characteristics

**Fig. 1:**
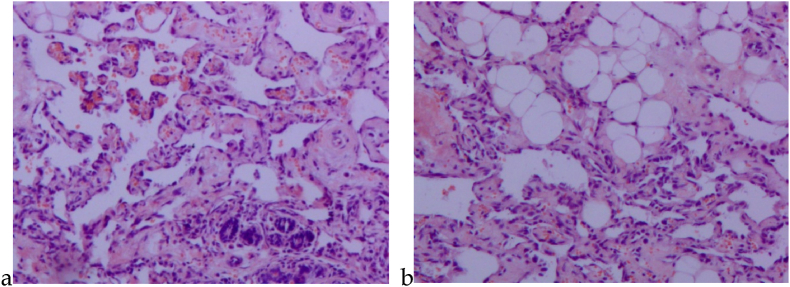
Vasogenic tumor; In view of the special location of the tumor, angiosarcoma was not excluded (hematoxylin and eosin, 100 × ). (a) Tumor cells infiltrating breast tissue and invading lobule tissue; (b) Tumor cells invading adipose tissue. In clinical practice, when encountering a pathological report indicating breast vasogenic tumor, we should be suspicious of angiosarcoma.

Based on Donnell et al.'s classification, BA pathology can be divided into three levels according to the degree of differentiation [12](Table 1). However, since the proposal of the self-organizing histologic grading, several scholars have studied its relationship with prognosis, and the results are controversial [[13], [14], [15]]. Recently, Maria G. Kuba et al. introduced a new 2-level grading system, grouping low and intermediate-grade angiosarcomas together (Table 1). The study aimed to reevaluate the impact of histologic grading as the primary factor, among several clinical-pathological variables, on prognosis. The research confirmed a correlation between prognosis and histologic grading in primary breast angiosarcoma. Nevertheless, the impact of this grading system on secondary breast angiosarcoma may be limited [16]. In clinical practice, there is a significant similarity in the description between well-differentiated angiosarcoma and vascular neoplasms. Due to the varied morphology of BA and the potential morphological resemblance between highly differentiated angiosarcoma and benign hemangioma, along with the rarity of benign breast hemangioma and the unfavorable prognosis associated with angiosarcoma, when presented with a pathology report indicating a breast vasogenic tumor, suspicion of angiosarcoma should be warranted [17](Fig. 1a and b). Furthermore, immunohistochemistry plays a crucial role in the diagnosis and differential diagnosis of angiosarcoma [18]. Research suggests that CD31 and CD34 are sensitive markers for endothelial cells, with CD31 identified as the most sensitive and specific endothelial cell marker. Ulex europaeus agglutinin-1 (UEA-1) is commonly utilized as an additional endothelial cell marker, exhibiting positive expression in vascular sarcomas but with reduced specificity. Factor VIII-related antigen serves as an endothelial cell marker, displaying positivity rates in vascular sarcoma cells ranging from 40 % to 100 %, thus offering diagnostic value for this condition [19]. ETS-related gene (ERG) and Friend leukemia integration 1 (FLI-1) emerge as novel markers for the identification of breast vascular sarcoma, showcasing heightened sensitivity and specificity compared to CD31 and CD34 [20].

**Table 1:** The histologic grading and features of breast angiosarcoma.

Histologic Features	Donnell, RM et al.			Kuba, Maria G et al.	
	GroupⅠ（Low）	GroupⅡ（Intermediate）	GroupⅢ（High）	Low	High
Lesion involves breast parenchyma	Present	Present	Present	Present	Present
Interanastomosing vascular channels	Present	Present	Present	Present	Present
Hyperchromatic endothelial cells	Present	Present	Present	Present	Present
Endothelial tufting	Minimal	Present	Prominent		
Papillary formations	Absent	Focally present	Present	Present	Present
Solid and spindle cell foic	Absent	Absent or minimal	Present	≤10 % of tumor area	＞10 % of tumor area
Mitoses (/mm2)	Rare or absent	Present in papillary areas	Numerous	0–10	Numerous
“Blood lakes”	Absent	Absent	Present	Present	Present
Necrosis	Absent	Absent	Present	Absent	Present

Note: In the classification by Kuba, Maria G et al., the following changes have been made. ①The term “papillary formations” is used exclusively; the term “endothelial tufting” is no longer employed. ②“Solid and spindle cell foci” has been replaced with “solid foci.” Solid lesions are categorized as focal (≤10 % of the tumor) or diffuse (>10 %). ③Semi-quantitative methods for mitotic count have been replaced with quantitative methods. Mitotic counts are classified into 3-tier system: score 1 (0–5 mitoses/mm^2^), score 2 (6–10 mitoses/mm^2^), and score 3 (＞10 mitoses/mm^2^). ④Papillary structures and “blood lakes” are applicable to all tumor grades.

## 4 Imaging findings

Breast X-ray imaging lacks specificity in detecting angiosarcoma. X-ray examinations often manifest as masses or asymmetric shadows. If there are calcified areas, they often lack features indicative of malignancy. Approximately one-third of patients may experience incorrect negative results in molybdenum target examinations, possibly due to the dense glandular structure [6]. In contrast, BA is characterized by vascular lumens, low cell density, and growth along the interface between fat and glandular tissue, lacking clear boundaries [21]. Ultrasound evaluation of angiosarcoma lacks distinct features, with rarely observed typical mass effects characterized by a gradual transition of borders with the surrounding normal tissue. It can present as hypoechoic, hyperechoic, or heterogeneous masses with posterior acoustic shadowing [1]. Contrast-enhanced ultrasound, a real-time, continuous imaging technique, observes the complete process of tumor microcirculation perfusion, assisting in diagnosing and distinguishing breast lesions. However, there is no literature reporting on the contrast-enhanced ultrasound characteristics of BA. Research by Yang OO et al. suggests that combining contrast-enhanced ultrasound and MRI to portray the augmented morphological and hemodynamic attributes of angiosarcoma can aid in preoperative diagnosis [22]. Breast MRI can uncover internal heterogeneous signals within the mass, displaying uneven enhancement on contrast-enhanced scans, indicating abundant vascularity within the lesion [23]. As a result, in comparison to other methods, breast MRI proves a more dependable non-invasive approach for detecting BA. MRI has demonstrated its capability to identify lesions that breast X-ray imaging might overlook [24]. BA lacks clear specificity in clinical presentation and imaging features, making it difficult to differentiate from conditions such as breast cancer, benign breast tumors, chronic inflammation, or breast hyperplasia [25]. Thus, the imaging portrayal cannot function as a conclusive diagnostic foundation; the ultimate diagnosis relies on pathological and immunohistochemical findings.

## 5 Selection of surgical approach

Currently, there is a lack of clear guidelines for the surgical management of BA. In general, a more rational surgical approach is often regarded as opting for a simple mastectomy. Expanding the scope of surgical resection or performing axillary lymph node dissection is not recommended. Achieving complete surgical resection with optimal margins serves as the foundation of surgical treatment [26]. While the optimal surgical approach remains unclear, achieving a negative margin in final surgical resection remains a primary treatment modality. Despite the high malignancy of BA, its involvement of regional lymph nodes is rare due to its origin in interstitial tissue. In a study by Rosen et al. involving 63 PBA patients from the Sloan Kettering Cancer Center, only one out of 35 patients who underwent axillary lymph node dissection exhibited metastasis [27]. As such, it is typically not advised to perform routine axillary lymph node dissection unless the presence of axillary lymph node metastases is confirmed, which may then necessitate a modified radical mastectomy. Reports suggest that total mastectomy offers the most effective surgical resection method [28]. Toesca and others argue that postoperative survival rates do not significantly differ between patients undergoing simple or radical mastectomy for BA [29]. Additionally, Han Qiong et al. found no statistically significant difference in survival rates among the breast enlargement excision group, mastectomy group, and breast-conserving surgery group through survival analysis and Cox multivariate analysis [30]. Furthermore, Ma Rong et al. highlight that a comprehensive and thorough resection during the initial surgery is an effective local control measure. For the first therapeutic surgical intervention, choosing mastectomy, ideally involving the pectoralis major and minor muscles, prevents the need for reoperation in case of recurrence [31]. In clinical practice, the selection of surgical approaches requires a comprehensive consideration of factors such as tumor size, pathological type, and patient preferences. Surgical excision and reconstruction necessitate collaborative multidisciplinary treatment involving breast surgeons, radiologists, medical oncologists, and plastic surgeons.

## 6 Postoperative adjuvant therapy

Postoperative adjuvant treatments for BA include radiotherapy, chemotherapy, and others, with no standardized protocol currently established [32]. Adjuvant therapy has the potential to enhance survival rates, but certain studies indicate a lack of benefit [3]. Therefore, the standard adjuvant treatment regimen for breast angiosarcoma patients is not yet clear, and further research is needed to determine the specific treatment outcomes.

Consensus on the benefits of adjuvant radiotherapy remains absent. In Sher et al.'s study, 68 % of patients received radiation, resulting in 5-year and 10-year relapse-free survival rates of 47 % and 44 %, respectively. These outcomes demonstrated prolonged relapse-free survival compared to patients without radiotherapy (33 % at 5 years and 25 % at 10 years) [33]. Despite these encouraging results, due to the limited number of participants, no significant association emerged between adjuvant radiotherapy and improved survival. Han Qiong et al.'s research involving 34.3 % of PBA patients receiving radiotherapy exhibited survival curve outcomes that showed no significant link between radiotherapy and patient prognosis, leading to non-recommendation of radiotherapy as a routine postoperative adjuvant therapy [30]. Nonetheless, some radiologists contend that adjuvant radiotherapy following surgery is advantageous for breast sarcomas, particularly in cases with microscopically positive margins [34]. However, whether radiation therapy can be conducted remains a question, as SBA is induced by previous radiation therapy. Additionally, reports have emerged on proton beam radiotherapy for BA postoperative treatment [35]. Hyperfractionated radiotherapy demonstrates benefits for local control and survival rates [3].

The efficacy of adjuvant chemotherapy for BA remains uncertain after numerous years, and there is no international consensus on chemotherapy regimens for these patients. Studies suggest that docetaxel profoundly inhibits hemangiosarcoma proliferation, and paclitaxel-dominated chemotherapy can enhance survival rates [36]. Hirata et al. showcased improved overall survival in 41 metastatic hemangiosarcoma patients treated with taxane [37]. Tierney et al. validated that anthracycline-based chemotherapy significantly enhanced disease-free and overall survival [38]. Stacchiotti et al. identified that gemcitabine extended progression-free survival in advanced BA patients [39]. Mahdi et al.'s data imply the effectiveness of actinomycin D in some BA patients [40]. Moreover, several reports suggest that beta-blockers, particularly propranolol, substantially decrease tumor proliferation, contributing to BA treatment [41]. In recent years, with the in-depth research and development of vascular endothelial growth factor (VEGF-A), VEGF-C, and their receptor VEGF-R1, anti-angiogenic therapy has emerged as a highly promising treatment method. This approach can inhibit the formation of new blood vessels, thereby reducing the blood supply to tumors, decreasing the delivery of oxygen and nutrients, and consequently restricting tumor growth [42]. Additionally, clinical trials are underway for vascular sarcoma involving the use of Pamulizumab in combination with targeted drugs such as Axitinib [43]. Despite the lack of compelling data supporting adjuvant chemotherapy regimens, for metastatic or locally advanced diseases, chemotherapy based on Doxorubicin remains the frontline standard treatment for unresectable or metastatic angiosarcoma, exhibiting a progression-free survival of 3.7–5.4 months [44]. Nevertheless, in some experiences, weekly Paclitaxel has demonstrated good tolerability and activity [45]. For recurrent BA, studies have indicated promising results with chemotherapy based on bleomycin. Additionally, electrochemotherapy has emerged as a novel option, particularly in recurrent cases, although more robust data are needed [6]. The results from whole exome sequencing (WES) on 47 samples, taken from a subset of 338 patients as part of the Angiosarcoma Project in the USA and Canada, reveal a higher frequency of PIK3CA mutations in breast cancer compared to other carcinomas. This implies that PI3Kα inhibitors, commonly employed for breast cancer treatment, may show potential for the future management of primary angiosarcoma of the breast [46].

## 7 Prognosis

The prognosis of BA is poor, with reported 5-year survival rates varying between 28 % and 54 % in the available literature. The average disease-free survival stands at 2.26 years, with an overall survival of 2.96 years [47]. A particular study revealed a higher mortality risk among patients diagnosed with SBA compared to those with PBA. This elevated risk is primarily attributed to older age, higher tumor grading, and advanced stage observed in SBA patients [48]. Within a systematic review, the 5-year interval of local recurrence-free survival for SBA was reported to be 32 % [49]. Meanwhile, distinct studies documented a median overall survival period following histological diagnosis ranging from 10.8 to 33.5 months [50]. In addition to frequent local recurrence, the occurrence of distant metastases is also noteworthy. Common sites implicated in such metastases include the contralateral breast, lymph nodes, lungs, pleura, bones, liver, and more distant skin areas. Although instances of metastasis to the orbit are rare, when contralateral breast involvement arises, it is often considered an expression of subcutaneous spread. Notably, research highlights the lungs and liver as the prevailing sites for distant metastatic occurrences [51]. Several adverse prognostic factors contribute to the unfavorable outlook of BA, encompassing advanced age, tumor size, and histological grading [3]. Furthermore, some studies suggest that the occurrence of pregnancy or lactation also factors into adverse prognoses [7].

## 8 Summary

In summary, BA is a clinically rare and highly malignant tumor with a poor prognosis. Both clinical and radiological presentations lack specificity, which can lead to misdiagnosis or missed diagnosis. Accurate diagnosis can only be achieved through thorough histopathological sampling coupled with immunohistochemical analysis to enhance diagnostic accuracy. Additionally, we speculate that the occurrence of BA might be associated with elevated estrogen levels. At present, standardized treatment guidelines for BA are absent, and treatment outcomes remain unsatisfactory. Surgery is the primary treatment approach, with achieving negative surgical margins serving as the foundation. Simple mastectomy is a reasonable surgical option, and the expansion of surgical resection or axillary lymph node dissection is generally not recommended. The role of adjuvant radiotherapy and chemotherapy is still debated, and further research is required to determine their specific therapeutic effects. Therefore, clinical practitioners should be well-versed in its clinical and pathological features, understand its diagnostic criteria, and strive for early diagnosis and treatment to extend patient survival and improve cure rates. Moreover, we recommend a multidisciplinary approach involving collaboration among breast surgeons, medical oncologists, radiologists, and plastic surgeons, as this can yield significant benefits and provide comprehensive care for patients' health and quality of life.

## Funding

This work was supported by Weifang Health and Family Planning Commission (wfwsjs_2018_029).

## Data availability statement

No data was used for the research described in the article.

## CRediT authorship contribution statement

**Ran An:** Writing – original draft, Methodology. **Xiao-Juan Men:** Supervision, Investigation. **Xi-Hao Ni:** Project administration. **Wei-Tao Wang:** Visualization. **Chang-Liang Wang:** Writing – review & editing, Conceptualization.

## Declaration of competing interest

The authors declare that they have no known competing financial interests or personal relationships that could have appeared to influence the work reported in this paper.
